# Blending Wastes of Marble Powder and Dolomite Sorbents for Calcium-Looping CO_2_ Capture under Realistic Industrial Calcination Conditions

**DOI:** 10.3390/ma14164379

**Published:** 2021-08-05

**Authors:** Paula Teixeira, Auguste Fernandes, Filipa Ribeiro, Carla I. C. Pinheiro

**Affiliations:** CQE—Centro de Química Estrutural, Instituto Superior Técnico, Universidade de Lisboa, Av. Rovisco Pais 1, 1049-001 Lisboa, Portugal; auguste.fernandes@tecnico.ulisboa.pt (A.F.); filipa.ribeiro@tecnico.ulisboa.pt (F.R.); carla.pinheiro@tecnico.ulisboa.pt (C.I.C.P.)

**Keywords:** CO_2_ capture, Ca-looping, sorbents, waste marble powder, dolomite, realistic conditions

## Abstract

The use of wastes of marble powder (WMP) and dolomite as sorbents for CO_2_ capture is extremely promising to make the Ca-looping (CaL) process a more sustainable and eco-friendly technology. For the downstream utilization of CO_2_, it is more realistic to produce a concentrated CO_2_ stream in the calcination step of the CaL process, so more severe conditions are required in the calciner, such as an atmosphere with high concentration of CO_2_ (>70%), which implies higher calcination temperatures (>900 °C). In this work, experimental CaL tests were carried out in a fixed bed reactor using natural CaO-based sorbent precursors, such as WMP, dolomite and their blend, under mild (800 °C, N_2_) and realistic (930 °C, 80% CO_2_) calcination conditions, and the sorbents CO_2_ carrying capacity along the cycles was compared. A blend of WMP with dolomite was tested as an approach to improve the CO_2_ carrying capacity of WMP. As regards the realistic calcination under high CO_2_ concentration at high temperature, there is a strong synergetic effect of inert MgO grains of calcined dolomite in the blended WMP + dolomite sorbent that leads to an improved stability along the cycles when compared with WMP used separately. Hence, it is a promising approach to tailor cheap waste-based blended sorbents with improved carrying capacity and stability along the cycles under realistic calcination conditions.

## 1. Introduction

The Paris Agreement signed in 2016 has the main target “to hold the increase of the global average temperature well below 2 °C above pre-industrial levels and to pursue efforts to limit the temperature increase to 1.5 °C above pre-industrial levels”[1]. The Global CCS Institute report concluded that the extra costs of CO_2_ capture and the absence of policies to justify investment are primary barriers to large-scale deployment of carbon capture and storage (CCS) in power generation, but good progress has been made to reduce the cost and to optimize the carbon capture technologies performance. Additionally, CCS is considered a key option available for deeply decarbonizing cement, steel and iron production [2].

Ca-looping (CaL) is one of the most promising second-generation technologies for the cement industry post-combustion CO_2_ capture, based on the reversible chemical reaction of CaO and CO_2_ to form CaCO_3_, but it still has some limitations related to the sintering of CaO sorbents and consequently with the decrease of the sorbents CO_2_ capture capacity during the cyclic operation. The selection of sorbents with high carrying capacity and stability is essential for the implementation of the CaL technology on a large scale [3]. Compared to other technologies, the CaL sorbent can be easily regenerated and, additionally, the initial CO_2_ capture efficiency is especially high during the initial carbonation–calcination cycles. During the calcination step, the CO_2_ can be selectively separated from CaCO_3_, and with a high CO_2_ partial pressure, a pure stream suitable for storage or for conversion processes can be generated. Furthermore, the CaL concept has unique potential advantages, i.e., it can be integrated into a variety of plants, and the exhausted sorbent (CaO) can be used as raw material in the cement plants, allowing the reduction of the carbon footprint of this industry that is responsible for 5% of anthropogenic global CO_2_ emissions [4,5,6]. Another advantage of CaL is the use of natural and non-expensive CaO-based sorbents, such as limestone, which is a resource widely distributed and available on the Earth’s surface.

Natural limestone (CaCO_3_) is the perfect candidate for CO_2_ capture, but its deactivation along the cycles under calcination temperatures higher than 800 °C is significant, and the CaO conversion usually reaches very low values in the range 20–30% after a few cycles [7]. Several efforts have been made to understand the mechanisms associated with the CaO-based sorbents’ deactivation to increase their lifetime [8,9]. This loss of capacity is mainly attributed to the sorbents sintering and pore blocking. The sorbent sintering is correlated with the loss of micropores and mesopores and crystallite size growing along the cycles, causing surface area and total pore volume reduction and changes in the pore size distribution [10,11,12,13]. Some authors believe that the average pore size overrides the contribution of the surface area [14,15]. In fact, dynamic simulations evidence that particles with a higher separation distance have a larger CO_2_ uptake due to a lower occurrence of sintering [16]. Strategies for lifetime enhancement of CaO-based sorbents include: (1) surface modification using solid supports such as inert porous alumina [8]; (2) use of additives or dopants to obtain CaO-based mixed oxides with higher stability [10,17,18,19]; (3) changing the morphology and microstructure, e.g., preparation of nano-particles [10,19]; (4) use of different synthetic precursors with a rich microporous structure [10,18,20,21,22,23,24].

In addition to the advantages of synthetic and modified sorbents, at the industrial level, their use is limited due to the associated energy costs. A compromise should be taken between the sorbent performance, cost and environmental issues, so practical, scalable and inexpensive CaO-based sorbents should be used [15,25]. To promote the industrial CaL process development and to move on to the next technology readiness level (TRL) step, it is necessary to guarantee the sustainability of this technology focusing on economic and ecological materials, i.e., by using an eco-friendly approach.

Due to environmental issues, several solid CaO sorbents recovered from waste resources have been tested in the CaL process: WMP [15,26,27], carbide slag [28,29], blast furnace [30], eggshells, shellfish shells and cuttlefish bones [27,31,32,33,34,35] and paper and pulp industry sludge [36]. Additionally, to improve the sorbents’ stability and reactivity along the cycles, some wastes were also tested as supports or doping materials: bottom and fly ash [36,37,38,39,40,41,42], biomass ash [41], industrial sludge [42], spent blenching clay [43] and spent fluid catalytic cracking catalyst (FCC) [36]. Among the CaCO_3_-based wastes already proposed for CO_2_ capture, the WMP composed mainly of CaCO_3_ and vestigial amounts of other elements (Mg, Si, Al, Fe, etc.) is one of the most promising wastes for CaL since it presents a better performance for CO_2_ capture than limestone. Pinheiro et al. [15] showed that after 10 cycles, the CaO conversion of WMP was higher than for the case of limestone, i.e., it was ca. 47–57% and 30% for WMP and limestone, respectively.

After limestone, dolomite is the most abundant carbonate in Earth’s crust, and it contains calcium and magnesium carbonates in different proportions, Ca_(1-x)_Mg_x_(CO_3_)_2_. This mineral has been identified as an adequate precursor for CO_2_ capture, but the role of MgO in CO_2_ capture needs to be better understood. Thermodynamic data indicate that for temperatures between 600 and 700 °C and CO_2_ partial pressures in the range 0.15 to 0.30 bar used in the carbonation step of CaL process, MgO is almost inert, i.e., it does not capture CO_2_ [44,45], but previous studies evidenced a higher CO_2_ carrying capacity and stability for dolomite than for limestone. The presence of chemical elements such as Mg, Al and Si, during the carbonation–calcination cycles influences the skeleton/microstructure of the calcined sorbent, which hinders aggregation or sintering of CaO crystallites and helps in preserving the nanocrystalline CaO structure [46]. Some approaches have been carried out to improve the CO_2_ capture by dolomitic carbonates, such as acid acetic pre-treatment [47] and two-step calcination under N_2_ and CO_2_ atmosphere to modify the sorbent microstructure [48], and ball milling of dolomite [49], even with water [50] or with ice and water to regenerate the dolomite and increase the CO_2_ carrying capacity through the elimination of CaO and MgO segregation after cyclic carbonation–calcination cycles [51]. However, more detailed studies are still necessary to understand the role of MgO on the enhancement of the stability and multicycle carrying capacity performance observed for this alternative natural CaO-based sorbent, especially under a realistic industrial calcination gas atmosphere with a high concentration of CO_2_.

For the storage or downstream utilization of captured CO_2_, the sorbents’ calcination step should be conducted under realistic and convenient gas atmospheres with a high concentration of CO_2_, which implies the use of calcination temperatures higher than 900 °C due to the reaction thermodynamic equilibrium. The use of concentrated CO_2_ atmospheres approximating realistic industrial CaL calcination conditions for generating an exit gas stream with a high concentration of CO_2_ is still a challenge [3], and the sorbents are rarely tested using laboratory scale reactors under severe regeneration calcination conditions. In a recent review focused on the development of sorbents for CaL, only ~6% of experimental tests were performed under high CO_2_ concentration using laboratory scale reactors [52]. In our previous work, it was shown that WMP are promising waste materials to be used as sorbents for CaL, but the calcination was carried out under a mild calcination atmosphere (N_2_, 800 °C). Therefore, this work has the main goal of filling this gap for testing and comparing the performance of natural CaO-based sorbents, such as WMP, dolomite and their blend, for cyclic CaL CO_2_ capture using a calcination atmosphere with a realistic high concentration of CO_2._ The CaL tests were carried out in a fixed bed reactor using two different calcination conditions: (a) at 800 °C under 100% N_2_ atmosphere, which will be considered as “mild” calcination conditions in this work; and (b) at 930 °C under a gas atmosphere of 80% CO_2_ balanced in N_2_, which will be called as “realistic” calcination conditions in this work. A blend of WMP with dolomite was also tested as an approach to improve the WMP sorbents carrying capacity and stability.

## 2. Materials and Methods

### 2.1. Materials

Samples of WMP and dolomite, both with particles lower than 63 µm, were used as CaO sorbent precursors. The WMP sorbent was collected in a Portuguese marble producer plant in the region of Estremoz (Portugal), and the dolomite sample (Omyadol SF-GZ) is from Turkey. A sample of a blended sorbent with 80% of CaO and 20% of MgO in a calcined basis (wt.%) was prepared by mechanical mixture of the adequate amounts of WMP and dolomite. The samples were dried and kept at 120 °C before the experimental tests to eliminate the moisture.

### 2.2. Characterization Methods

The elemental composition of WMP and dolomite was determined at LAIST (Laboratory of Analysis of Instituto Superior Técnico). Calcium, magnesium, aluminum, silicon, and iron were analyzed by inductively coupled plasma–optical emission spectrometry, and the carbon was determined by an internal method (accredited by the Portuguese Institute of Accreditation). The elemental composition is essential to estimate the CO_2_ capture capacity of the CaO-based sorbents along the carbonation–calcination cycles.

The BET specific surface area (S_BET_) and the pore size distribution (PSD) of the used sorbents after n cycles (n = 0, 2, 5, 10, 20 cycles) of calcination–carbonation were assessed by N_2_ sorption at −196 °C on a Micromeritics ASAP 2010 apparatus. Before the analysis, the samples were outgassed under vacuum at 90 °C for 1 h and then at 350 °C for 5 h. The total pore volume (V_p_) was calculated from the adsorbed volume of nitrogen for a relative pressure (P/P_0_) of 0.97. The BET equation was applied to estimate the S_BET_, and the PSD distribution was achieved by using the BJH model (desorption branch).

The macropores size distribution of the selected sorbents was determined by Hg porosimetry, using a Micromeritics Autopore IV 9500. During the test, the pressure range varied between 0.5 and 30,000 psi and a contact angle of 130° was considered.

In order to identify the different crystalline phases, powder X-ray diffraction (PXRD) diffractograms of fresh and used sorbents were obtained using a D8 Advance X-ray diffractometer (Bruker AXS GmbH, Karlsruhe, Germany) using Cu Kα (λ = 0.15406 nm) radiation operating at 40 kV and 40 mA. The measurements were made between 15 and 70° in 2θ, with a step size of 0.03° and step time of 3 s. The crystallography open database (COD) was used to identify the crystalline phases, and the TOPAS 4.2 software (Bruker) was used to quantify the amount of the different phases by means of Rietveld refinement. The crystallite size of sorbents was estimated using Scherrer’s equation (D = Kλ⁄(b cos θ)), based on the XRD data. D is the crystallite size (nm), b is full width at half maximum (FWHM) of the XRD peak considered, λ is the wavelength (0.15406 nm), θ is the Bragg angle (degree) and K is Scherrer´s constant (K = 0.9, assuming that the particles are spherical).

### 2.3. Carbonation–Calcination Tests at In Situ XRD Chamber

**Figure 1:**
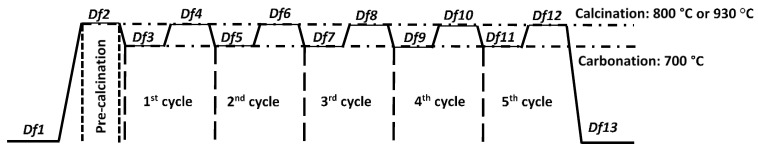
In-situ XRD carbonation–calcination cycles procedure.

In situ XRD carbonation–calcination cycles were carried out in a Bruker D8 Advance X-ray diffractometer equipped with an Anton Paar HTK 16N High Temperature Chamber. The carbonation was performed at 700 °C and calcination at 800 or 930 °C, for mild or realistic calcination conditions, respectively. The measurements were made between 28 and 39° in 2θ for WMP, dolomite, and for the blended sorbent. The CaCO_3_ peaks (29.3° and 35.9°) and CaO peaks (32.3° and 37.5°) profiles were evaluated. A step size of 0.05° and a step time of 1 s was used for all samples. A first diffractogram (Df1) was carried out at room temperature and after each carbonation or calcination gas–solid reaction at high temperature. The carbonation or calcination reactions were carried out for 10 min, and after this time, the XRD diffractograms (Dfn) were obtained. Figure 1 shows the scheme of the procedure used. A flow of 100 mL was used along the carbonation–calcination cycles, 15% of CO_2_ balanced in N_2_ during the carbonation, and 100% of N_2_ or 80% of CO_2_ balanced in N_2_ for mild and realistic calcination conditions, respectively. The heating temperature rate between 700 and 800/930 °C was 20 °C/min while the cooling temperature rate between 800/930 °C and 700 °C was 50 °C/min.

The recorded XRD diffractograms were analyzed using TOPAS 4.2 software, and the relative amounts of CaCO_3_ and CaO, based on the patterns obtained in the range 28–39° in 2θ, were quantified by means of Rietveld refinement.

### 2.4. Carbonation–Calcination Tests in a Fixed Bed Reactor

The cyclic carbonation and calcination reactions were carried out in an experimental laboratory-scale fixed-bed reactor system under mild (800 °C, 100% N_2_) and realistic (930 °C, 80% of CO_2_ balanced in N_2_) calcination conditions. The realistic calcination conditions were selected based on the CO_2_ partial pressure equilibrium diagram [53]. For an atmosphere of 100% of CO_2_, the equilibrium temperature is ca. 900 °C, so the calcination occurs very slowly at this temperature, which is not adequate for industrial applications. Therefore, a calcination temperature higher than the equilibrium temperature should be used. Indeed, the use of 80% of CO_2_ and calcination temperatures around 930 °C were selected to allow reducing the time needed for the complete calcination and simultaneously to avoid severe sintering. The experimental system consists of a gas feeding system, a reactor system with temperature control, and a CO_2_ gas analyzer. The unit includes an oven and a quartz reactor with an internal diameter of 0.03 m and a length of 0.10 m and a porous plate to support the adsorbent. A stream of CO_2_ and N_2_ (for carbonation and realistic calcination conditions) or only N_2_ (for mild calcination conditions) was fed to the quartz reactor, and the flows were controlled with Alicat and Brooks mass flowmeters, for N_2_ and CO_2_, respectively. During the carbonation–calcination cycles, the CO_2_ gas concentration at the outlet stream was measured with a Guardian Plus equipment in the range 0–30 ± 0.75% (mild calcination conditions) or 0–100 ± 2% (realistic calcination conditions). The oven temperature was controlled by an Eurotherm^®^ 2000 (Eurotherm Limited, Faraday Close, Worthing, United Kingdom) series equipment, and the temperature inside the quartz reactor was monitored by a thermocouple type K. The Labview software interface was used for data acquisition.

The sorbent sample (2 g) was loaded into the reactor and pre-calcined at 800 °C under N_2_ atmosphere until the CO_2_ release stopped (mild calcination conditions) or 930 °C for 15 min under 80% of CO_2_ balanced in N_2_, followed by N_2_ atmosphere until the CO_2_ release stopped for ensuring the complete sorbent decarbonation. After this activation step, the temperature was cooled down to 700 °C, and the carbonation step was conducted with a feed stream of 15% *v/v* of CO_2_ (150 mL/min of CO_2_ balanced in N_2_, to simulate a realistic CO_2_ concentration in the flue gases) until stabilization of the outlet stream CO_2_ concentration, which means that the sorbent reached its maximum CO_2_ capture capacity. After carbonation, the CO_2_ feed flow was stopped, and the sample was heated up to 800 °C under pure N_2_ flow (mild calcination conditions) or heated up to 930 °C for 15 min, under 80% of CO_2_ balanced in N_2_ (realistic calcination conditions) followed by a pure N_2_ flow. Then the sample was cooled down to 700 °C for a new carbonation–calcination cycle. To determine the textural and structural changes of CaO based sorbents, experimental CaL tests were performed with a different number of cycles (e.g., after 2, 5, 10 and 20 cycles), and the calcined sorbents were collected and immediately analyzed by N_2_ sorption and XRD techniques. For comparative reasons, an experimental test only including the pre-calcination (0 cycles) was performed under mild calcination conditions, i.e., after the sorbent activation, the experiment was stopped, and the calcined sorbent was collected and characterized.

The amount of CO_2_ captured (moles) in each carbonation step, $n_{{\mathrm{CO}}_{2},\mathrm{carb}},\;$was estimated by Equation (1):

(1) $$n_{{\mathrm{CO}}_{2},\mathrm{carb}}=\int_{t1}^{t2}\left(n_{{\mathrm{CO}}_{2},i}-n_{{\mathrm{CO}}_{2},\mathrm{not}\;\mathrm{capt}}\right)\mathrm{dt}\;\;\;\;$$

where $n_{{\mathrm{CO}}_{2},i}$ and $n_{{\mathrm{CO}}_{2},\mathrm{not}\;\mathrm{capt}}$ are, respectively, the molar amount of CO_2_ fed to the reactor and the molar amount of CO_2_ gas that did not react, measured in the off gas during carbonation, between carbonation time *t*1 and *t*2. The CaO conversion along the carbonation–calcination cycles was determined by Equation (2):

(2) $$\mathrm{CaO}\;\mathrm{conversion}=\frac{n_{{\mathrm{CO}}_{2},\mathrm{carb}}\times {\;M}_{\mathrm{CaO}}}{m_{\mathrm{sorbent}}\times {\;w}_{\mathrm{CaO}}}\times 100\left(\%\right)$$

where${\;M}_{\mathrm{CaO}}$ is the molar mass of CaO, $m_{\mathrm{sorbent}}$ is the initial mass of the sorbent and $w_{\mathrm{CaO}}\;$is the percentage of CaO in the initial mass of the sorbent. The CaO content was estimated through elemental chemical analysis of the fresh materials.

## 3. Results

### 3.1. Sorbent’s Characterization

The chemical oxide composition of the fresh WMP and dolomite sorbent samples, dried at 120 °C, is presented in Table 1.

Table 1 shows that the WMP, such as the limestone, is mainly composed of CaO and CO_2_. ASTM C119-16 [54] classifies limestone and marble in different groups of rocks, marble is a carbonate rock that has acquired a distinctive crystalline texture by recrystallization, usually due to high heat and pressure during metamorphism, and is mainly composed of the carbonate minerals calcite and dolomite, individually or in combination. Dolomite belongs to the limestone group and, besides CO_2_, contains two main oxides in its composition: CaO and MgO. A ratio of Ca/Mg (wt.%) of 2.4 was obtained, so this sorbent is classified as a calcitic dolomite. Compared to the WMP, lower amounts of Si, Al and Fe were found in dolomite.

**Figure 2:**
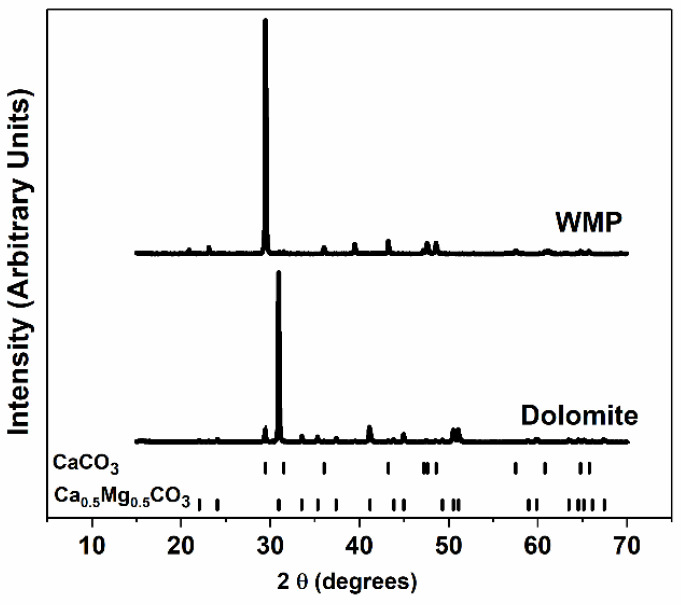
XRD patterns of the fresh sorbents dried at 120 °C.

XRD measurements were performed to identify the sorbent phases. The XRD diffractograms of the fresh sorbents dried at 120 °C are presented in Figure 2. CaCO_3_ is the main compound of WMP, which is in agreement with its chemical composition. For dolomite, the main phase is Ca_0.5_Mg_0.5_CO_3_ (78%), followed by CaCO_3_ (22%), which evidences that besides the dolomite phase, this sorbent also contains some isolated calcite (CaCO_3_).

### 3.2. Evaluation of Sorbents Reactivity by In-Situ XRD

The carbonation–calcination cycles using WMP and dolomite as sorbents were performed within an in-situ XRD chamber, and the evolution of CaCO_3_ and CaO crystalline phases present in each one of the materials samples was recorded in the XRD diffractograms in the range 28–39° in 2θ.

**Figure 3:**
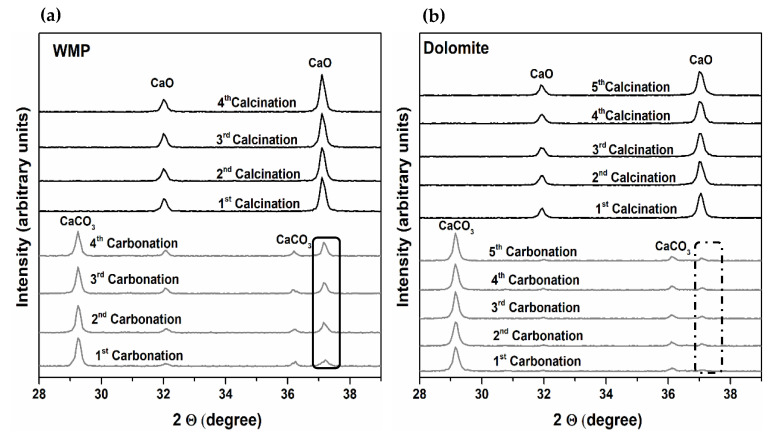
in-situ XRD diffractograms of WMP (**a**) and dolomite (**b**) obtained along the carbonation–calcination cycles: carbonation with 15% of CO_2_ at 700 °C and calcination with 100% of N_2_ at 800 °C.

Figure 3a,b show results obtained when the calcination was performed under N_2_ at 800 °C. In the case of WMP (Figure 3a), the relative amount of CaO that is not converted to CaCO_3_ at the end of the carbonation stage increases with the number of carbonation cycles (black rectangle line). However, in the case of dolomite (Figure 3b), the amount of CaO that is not carbonated after the carbonation stage (dotted line rectangle), though increasing, is less significant.

For evaluating the effect of the calcination temperature on the activity of WMP and dolomite sorbents, the calcination steps were performed under mild and realistic calcination conditions. The amount of unreacted CaO during carbonation, based on the relative amount of CaCO_3_ and CaO, was obtained from Rietveld refinement for both temperatures, considering only CaO and CaCO_3_ phases between 28 and 39° in 2θ. 

**Figure 4:**
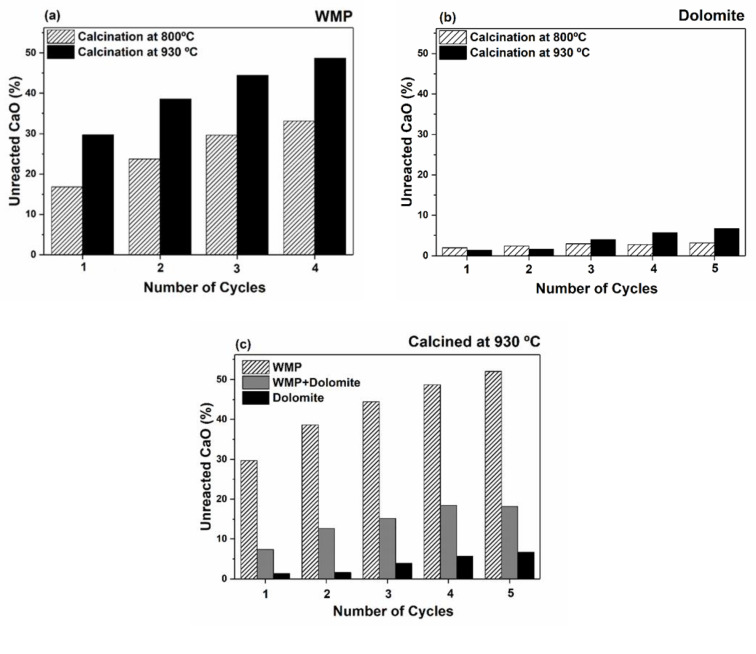
Amount of unreacted CaO (%) during the carbonation step with 15% of CO_2_ at 700 °C, and calcination with 100% of N_2_ at 800 °C (mild conditions) or 80% of CO_2_ at 930 °C (realistic conditions): (**a**) WMP, (**b**) dolomite; and (**c**) WMP, dolomite and a blend of WMP and dolomite with 80% of CO_2_ at 930 °C.

As shown in Figure 4a and 4b, the amount of unreacted CaO during the carbonation step, increases for both sorbents when a higher calcination temperature is used, confirming that the temperature increase from 800 to 930 °C favors the sorbents loss of activity. In the case of dolomite, during the first and second cycles, the unreacted CaO is similar for both temperatures, which can be justified by the lack of accuracy near XRD quantification limits. Considering the fourth carbonation as reference, the percentage of unreacted CaO between 800 and 930 °C increases 50 and 102% for WMP and dolomite, respectively, evidencing that the dolomite sorbent skeleton/microstructure suffers more changes in this range of temperatures than the WMP. Moreover, the fraction of unreacted CaO in dolomite during carbonation step is lower than in the case of WMP, meaning that the carbonation during the Ca-looping is more efficient for dolomite than for WMP, maybe because the MgO hinders the sintering of CaO [46].

Since the MgO present in the sorbent is expected to hinder the CaO sintering, a preliminary test was carried out at 930 °C with a blend of WMP and dolomite (80% CaO and 20% of MgO in a calcined weight basis) aiming to improve the WMP sorbent carrying capacity, and the results are compared with the individual sorbents. As can be observed in Figure 4c, blending WMP + dolomite seems to be a promising approach to improve the sorbents activity, since after five cycles, the amount of unreacted CaO in the blended sample is lower (19%) than in the WMP sample (52%). The results obtained by in-situ XRD studies are especially important to predict tendencies and compare sorbents’ carrying capacity.

### 3.3. Evaluation of Sorbents Reactivity for CO_2_ Capture under Different Calcination Conditions

**Figure 5:**
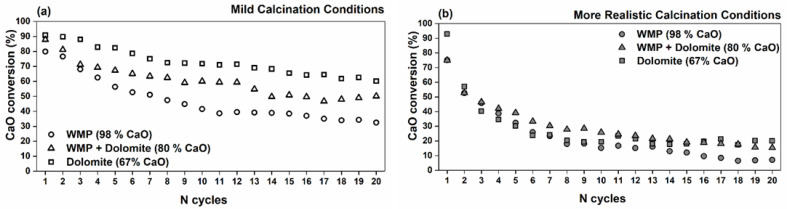
CaO conversion of WMP, dolomite and WMP + dolomite blended sorbent after 20 cycles of carbonation at 700 °C and calcination under (**a**) mild: 800 °C and 100% of N_2_, or (**b**) realistic: 930 °C and 80% of CO_2_ calcination conditions.

WMP, dolomite and a blend of WMP + dolomite sorbents were tested in a fixed bed reactor CaL unit under mild and realistic calcination conditions for evaluation of their cyclic CO_2_ capture performance. Figure 5 shows the CaO conversion (%) for 20 carbonation/calcination cycles for all the sorbents using mild (Figure 5a) or realistic (Figure 5b) calcination conditions. The sorbents’ CaO conversion was obtained based on the chemical composition of the sorbents (Table 1) and its CaO content because, under the used experimental conditions, MgO was shown to be inert [44].

At mild calcination (Figure 5a), dolomite sorbent with ca. 33% MgO (calcined basis) presents a higher initial CaO conversion on the first cycle, ca. 91%, and for WMP, it is ca. 80%, but after 20 cycles, the dolomite CaO conversion is approximately twice that of the WMP CaO conversion, i.e., 60 and 32%, respectively. The results show that the CaO content in the sorbent sample is not the most relevant factor, and an improved CaO conversion is achieved for dolomite sorbent due to the presence of other chemical elements (e.g., Mg).

A blend of WMP and dolomite (80% of CaO and 20% of MgO in calcined basis) was also prepared and tested for 20 cycles. An increase in CaO conversion was obtained with the increase of MgO content in the blended sorbent, i.e., the CaO conversion increased from 32% in WMP to 50% for the blended sorbent. The sorbents’ stability after 20 cycles (considering the first cycle CaO conversion as reference) was also evaluated, and the deactivation was 59, 41 and 34%, respectively, for WMP, WMP + dolomite blend and dolomite.

In the present study, the same three sorbents were also tested for 20 cycles under the above-mentioned realistic calcination conditions, and the CaO conversion (Figure 5b) was compared with the one attained under mild calcination conditions (Figure 5a). It is observed that the sorbents’ CaO conversion is much lower when realistic calcination conditions are used, but the three sorbents present a similar performance along the first 10 cycles. Figure 5b shows that in the case of realistic calcination conditions, dolomite achieved a CaO conversion of 20% after 20 cycles, which is higher than the corresponding CaO conversion observed for WMP + dolomite (15%) and for WMP (7%). Therefore, under realistic calcination conditions, the CaO conversion is much lower than under mild calcination conditions, but there is a stronger synergetic effect of inert MgO grains of calcined natural dolomite in the blend WMP + dolomite sorbents that leads to an improved stability along the cycles when compared with WMP used separately as sorbent.

### 3.4. Textural Properties of Sorbents Tested under Mild and More Realistic Calcination Conditions

**Figure 6:**
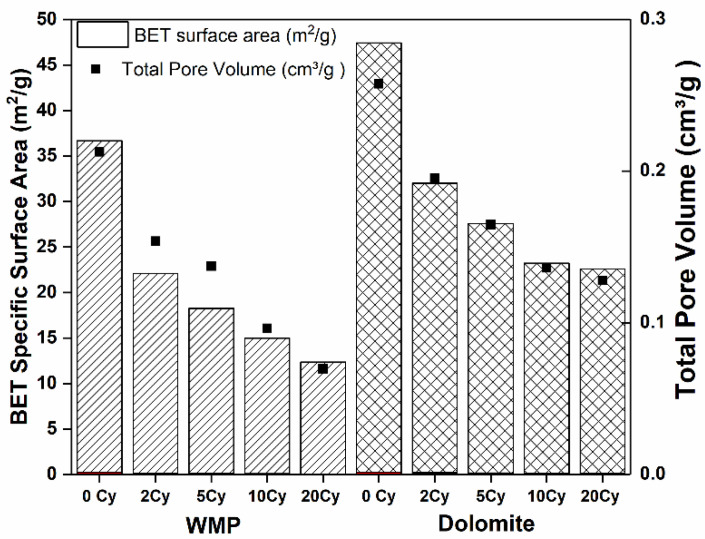
S_BET_ and V_p_ for WMP and dolomite samples after carbonation–calcination cycles under mild conditions.

The textural properties of the WMP and dolomite samples cyclically tested under mild calcination conditions were evaluated. The samples were collected from the fixed bed unit after n carbonation–calcination cycles (n = 2, 5, 10 and 20), and the corresponding textural properties were correlated with CaO conversion data. Due to the hygroscopic properties of CaO, which can be easily converted to Ca(OH)_2_ in the presence of atmospheric moisture, the used samples were removed from the reactor for characterization before cooling down to room temperature, avoiding changes in their composition and textural properties. S_BET_ and V_p_ were determined for both used sorbent samples, and the results are presented in Figure 6.

The S_BET_ reduction from the sorbent activation (0 cycles) to the 20^th^ cycle was 66 and 52% for WMP and dolomite, respectively. The higher initial values of S_BET_ and V_p_ and the lower reduction of S_BET_ of dolomite sorbent along the carbonation–calcination cycles can explain the CaO conversion results observed in Figure 5a. For both sorbents, the S_BET_ decreases along the cycles, as expected, but this decrease, due to the loss of particles porosity, is more significant for WMP than for dolomite in agreement with the higher deactivation rate of WMP (59%) comparatively with dolomite (34%) during the experiments carried out in the fixed bed. As reported in the literature [55], the higher stability of certain sorbents can be justified by the presence of additional oxides that act as inert supports and reduce the neck blockage associated with the sintering mechanisms. According to the Rule of Tammann, sintering due to lattice diffusion is generally observed at temperatures above 0.5 Tm, where Tm is the melting temperature (in Kelvin). Nevertheless, surface diffusion is already expected to occur above 0.33 Tm (Hüttig Temperature) [56]. For CaO, T_Tammann_ and T_Hüttig_ are 1170 and 679 °C, respectively, which means that the sintering process should occur essentially due to surface diffusion and could even start during the carbonation carried out at 700 °C. Ideally, to act as inert supports, the oxides present in the CaO-based sorbents should have a melting temperature higher than that of CaO. In the case of the oxides identified in the composition of calcined WMP and dolomite (Table 1), only MgO has a melting temperature higher than CaO, i.e., 2800 °C, which means that it can act as inert support suitable for this reaction. Therefore, the considerable amount of MgO (Table 1) in natural dolomite justifies the lower sintering of this sorbent. Hu et al. [55] studied several inert supports, and MgO-based supports were classified as good candidates to minimize the sintering process, as well as the Al_2_O_3_-based support materials. On the other hand, Fe_2_O_3_ and SiO_2_ should not be desirable, attending to their lower melting temperatures (respectively, 1565 and 1730 °C), which means that the T_Tammann_ of Fe_2_O_3_ is 646 °C and that of SiO_2_ is 729 °C.

**Figure 7:**
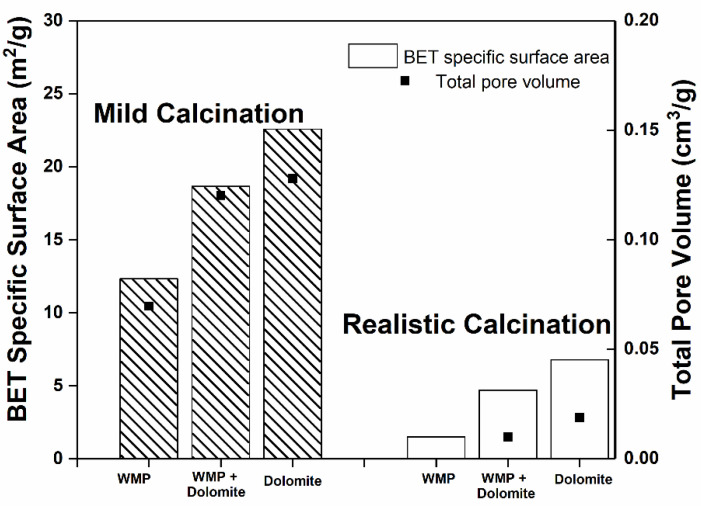
S_BET_ and V_p_ for sorbents WMP, dolomite and WMP + dolomite blended, after 20 cycles, under mild (800 °C and 100% of N_2_) and realistic (930 °C and 80% of CO_2_) calcination conditions.

The S_BET_ and V_p_ of sorbents WMP, dolomite and WMP + dolomite blend, tested under mild and realistic calcination conditions after 20 cycles, are shown in Figure 7.

Figure 7 shows that the calcination conditions during CaL cycles drastically affect the final S_BET_ and V_p_ of the sorbent samples. The S_BET_ of the three sorbents after 20 cycles decreases between 70 and 88% when comparing the mild with the realistic calcination conditions, thus explaining the results obtained for the CaO conversion decrease observed in Figure 5b. Taking into account that the T_Hüttig_ of CaO (679 °C) and MgO (741 °C) were largely exceeded, it can be considered that the sintering process due to solid-state diffusion that occurs in the calcination stage is enhanced at the higher temperature (930 °C) of the realistic calcination necessary to produce a concentrated stream of CO_2_ (80%).

Under mild calcination conditions, the WMP + dolomite blended sorbent (ca. 20% of MgO in the calcined sample) presents a higher S_BET_ and V_p_ than the WMP sorbent (ca. 1.1% of MgO in calcined sample); therefore, it can be seen that the high concentration of MgO in the blended sorbent favored an increase of ca. 51% of the S_BET_, in agreement with the higher CaO conversion observed in Figure 5a. Under realistic calcination conditions, the blended sorbent also presents a higher S_BET_ than WMP. It is observed that the S_BET_ of WMP decreases abruptly when the mild calcination conditions are replaced by the realistic ones, due to the low sintering resistance of this sorbent at higher temperatures.

**Figure 8:**
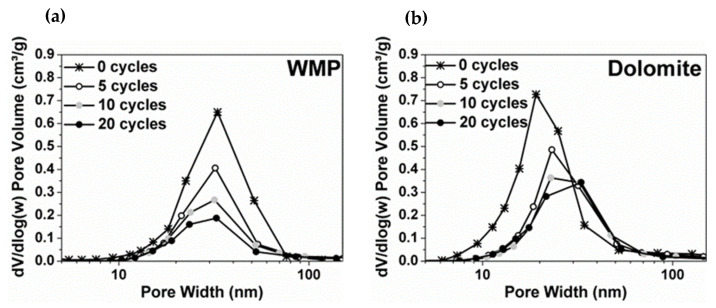
Pore size distributions obtained from the N_2_ adsorption–desorption technique (PSD from BJH desorption branch) for (**a**) WMP and (**b**) dolomite after 0, 5, 10 and 20 carbonation–calcination cycles under mild calcination conditions (800 °C and 100% of N_2_).

The pore size distribution (PSD) of sorbents tested under mild calcination conditions was estimated using BJH model (desorption branch of the isotherm). Figure 8 shows that the average pore width is mainly within the range of mesopores (2–50 nm). Dolomite has a Vp higher (0.72 and 0.30 cm^3^/g for 0 and 20 cycles, respectively) than WMP (0.64 cm^3^/g and 0.18 cm^3^/g for 0 and 20 cycles, respectively), which is in agreement with the higher CaO conversions of dolomite. Nevertheless, the average pore width of WMP particles is almost constant along the carbonation–calcination cycles (ca. 30 nm), while, for dolomite, the average pores width increases along the cycles. For 0 cycles, the average pore size was 19 nm, and then the smaller pores disappeared along the cycles and the average pore size became 30 nm after 20 cycles. The observed PSD profiles can be related to the sintering resistance of WMP and dolomite. In the case of WMP, the sintering of CaO particles causes mesopore volume reduction, without the formation of intermediate large mesopores. Besides the reduction of small mesopores and formation of intermediate size mesopores in dolomite along the carbonation–calcination cycles, after 20 cycles, this sorbent still maintains a higher mesopores content comparatively with WMP.

**Figure 9:**
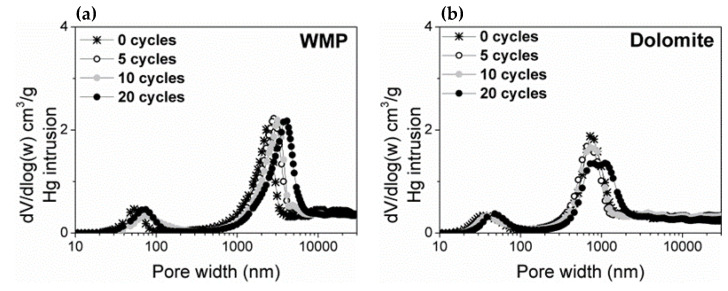
Pore size distributions obtained from the Hg porosimetry technique for (**a**) WMP and (**b**) dolomite after 0, 5, 10 and 20 carbonation–calcination cycles under mild (800 °C and 100% of N_2_).

The Hg porosimetry technique was also used to complete the average pore size distribution in the macropores region (Figure 9). The PSD curves show that WMP and dolomite present a bimodal pore size distribution with mesopores and macropores. Both sorbents show a slight increase of the average macropore width along the cycles, which can be related to the particles sintering, especially in the case of WMP. For similar Hg intrusion volumes, the average pore width for WMP is around 2200 nm, and for dolomite, it is around 800 nm, in agreement with the conversion profile for each sorbent, i.e., lower pore width contributes to a higher S_BET_ improving the CO_2_ capture capacity. 

**Figure 10:**
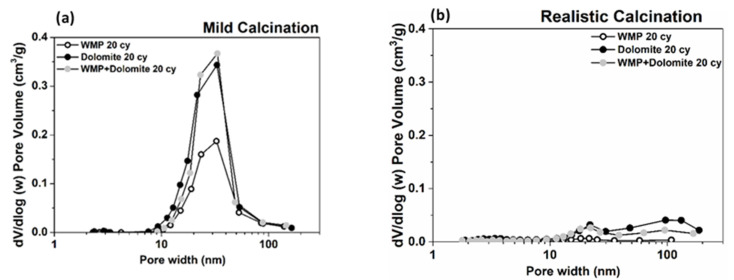
Pore size distributions obtained from the N_2_ adsorption–desorption technique (PSD from BJH desorption branch) for WMP, dolomite and WMP + dolomite mixed sorbent, after 20 cycles: (**a**) under mild (800 °C and 100% of N_2_) and (**b**) realistic (930 °C and 80% of CO_2_) calcination conditions.

Figure 10 compares the PSD curves of the sorbents tested under mild and realistic calcination conditions. The higher temperature of the realistic conditions results in a significant drop of the V_p_ content of all sorbents. At realistic calcination conditions, the dolomite sorbent V_p_ decreases significantly, but it maintains a small amount of mesopore volume content near an average pore size of 20–30 nm. The maintenance of mesopores volume is also observed in the blended sorbent that, due to some dolomite content, also presents content of mesopores slightly higher than WMP. These results can justify dolomite and the blended sorbents higher CaO conversion at realistic calcination conditions, showing that the S_BET_ should have a crucial role in CaO conversion (Figure 5).

**Figure 11:**
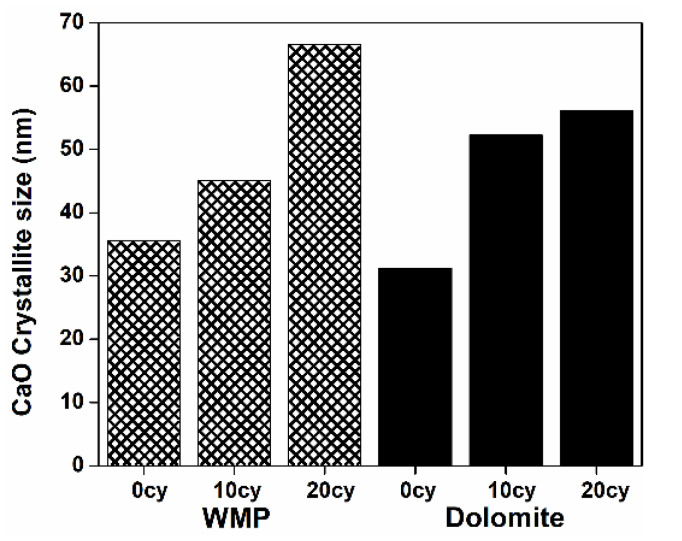
Average crystallite size (nm) for WMP and dolomite after 0, 10 and 20 carbonation–calcination cycles under mild calcination conditions (800 °C and 100% of N_2_).

The average crystallite size of the used sorbents was assessed by Scherrer’s equation from XRD data. Figure 11 shows that, even under mild calcination conditions, the average size of CaO crystallites increases 87 and 79% between 0 and 20 cycles for WMP and dolomite, respectively. Nevertheless, for dolomite, the average CaO crystallites size almost stabilized from the 10^th^ cycle on, maybe due to the presence of MgO that should limit the CaO crystallites growth. Indeed, between the 10^th^ and 20^th^ cycle, the crystallites size increases only ca. 7%.

A growth in the CaO crystallites size when the number of carbonation–calcination cycles increases was also observed by Biasin et al. [12], who verified that, by using a constant calcination temperature (900 °C), the CaO crystallites size increased 390% when the calcination time increased from 5 to 60 min.

**Figure 12:**
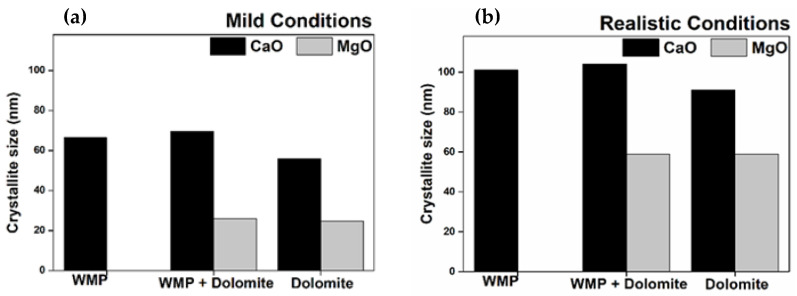
Average crystallite size (nm) for WMP, dolomite and WMP + dolomite mixed sorbent, after 20 cycles. (**a**) under mild (800 °C and 100% of N_2_) and (**b**) realistic (930 °C and 80% of CO_2_) calcination conditions.

Figure 12 shows that realistic calcination conditions lead to the increase of the average crystallites size of all sorbents. After 20 cycles, the CaO crystallite size increases 49, 52 and 62%, for WMP + dolomite blend, WMP and dolomite, respectively. For dolomite, the CaO crystallites size increase was more relevant because, under realistic calcination conditions, this sorbent partially loses the MgO protective effect that acts as a physical barrier between CaO particles. As previously explained, at 930 °C the T_Hüttig_ of MgO (ca. 741 °C) is largely exceeded. In the case of blended sorbent, the increase of CaO crystallite size was ca. 49%, which can be related to the lower MgO content in the blended sorbent than in dolomite (20% vs. 33%), so the loss of the MgO protective effect was less sharp in the blended sorbent, and consequently, the CaO crystallite size increase between the calcination temperatures of 800 and 930 °C was less crucial. This is in agreement with the results presented in Figure 4, where it can be seen that the dolomite sorbent skeleton/microstructure suffers more changes than the WMP between temperatures of 800 and 930 °C, which justifies the lower increase of the CaO crystallite size of WMP and of the blended sorbents.

Figure 12 also shows that the MgO crystallites were strongly affected by the calcination conditions, i.e., an increase in the average crystallites size of ca. 126% was observed when realistic calcination conditions were used. Anyway, the presence of MgO crystallites in the sorbent is still useful since they contribute to a higher separation between the CaO crystallites, hindering their sintering and allowing a lower CaO deactivation along the carbonation–calcination cycles (Figure 5).

Valverde et al. [57,58] studied in detail the correlation between the increase of CaO crystallites size and the CO_2_ partial pressures used in the calcination step. For CO_2_ partial pressures near the equilibrium (P/P_eq_ ~ 1), the calcination occurs slowly and a metastable CaO* phase contributing to the CaO nanocrystals agglomeration and crystallite size increase. In this case, the crystallites could reach the maximum value of 1000 nm [19], and the agglomeration is considered the main mechanism for the crystallites growth and sorbent deactivation. For low CO_2_ partial pressures (P/P_eq_ <<< 1), desorption and structural transformation occur rapidly, and the calcination rate is limited by chemical decomposition, so the metastable CaO* phase is present for very short times, hindering the growth of CaO crystallites by agglomeration. In that case, sintering is considered the main mechanism of crystallite growth, and usually the crystallites grow only until 50 nm. Besides the CO_2_ calcination partial pressure, the calcination temperature also has a relevant role in the CaO sintering. Abass et al. [59] evaluated the effect of CaCO_3_ decomposition temperature on the average CaO crystallites size and verified that it increased ca. 121–129% when the temperature increased from 800 to 1000 °C. This increase is higher than the one observed in Figure 12 for CaO crystallites; however, the range of calcination temperatures used in the present work is smaller (800–930 °C). For the case of the sorbents evaluated in the present work, the crystallites size increase should be explained by both factors: CO_2_ partial pressure and calcination temperature.

## 4. Discussion

The sorbent deactivation (Figure 5) is directly related to the MgO content (calcined basis) in the sorbents: 1% in WMP, 20% in the blended sorbent and 33% in dolomite. It is well known that MgO does not undergo carbonation under typical CaL conditions [45,46], but it provides additional pore volume during the dolomite calcination. Furthermore, the high Tammann temperature (1276 °C) for MgO allows the stabilization of the pore structure of dolomite [60]. It is generally accepted that carbonation of CaO grains occurs through two differentiated phases consisting of a fast reaction-controlled stage followed by a much slower solid-state diffusion stage. Some studies [47] have shown that the improved CO_2_ capture capacity of dolomite is mostly due to the greatly promoted carbonation in the solid-state diffusion phase. Solid-state diffusion is promoted by impurities and lattice defects; hence, the presence of MgO inert crystallites in calcined dolomite favors CaO carbonation in the solid-state diffusion-controlled phase. Therefore, at the usual non-realistic mild calcination conditions reported in the literature, the improved performance of dolomite with the increasing number of cycles, can be attributed to the stabilizing effect of the inert MgO skeleton, which hinders the aggregation and subsequent sintering of the CaO crystallites during the CaCO_3_/CaO transformation. Nevertheless, under realistic calcination conditions, the higher temperature that is required at realistic industrial calcination conditions, due to the high concentration of CO_2_ in the calciner, significantly affects the sorbents’ performance. Therefore, the expected realistic sorbents’ performance under industrial operating calcination conditions can be very different from the typical results found in the literature in experimental research tests using CaL sorbents, which are usually tested using mild calcination conditions under N_2_ or air atmospheres, not realistic from an industrial point of view and far from the real ones. This conclusion reinforces the need for more studies under real calcination conditions, i.e., under high CO_2_ concentration with different types of blends of solid wastes, such as wastes of marble powder and cheap natural dolomite to supply streams with high concentration of CO_2_ at the exit of the calciner adequate for subsequent CO_2_ conversion processes. The availability of WMP and dolomite as CaO (and MgO) precursors is not a limitation for their use as sorbents in the CaL process. The dolomite is the second most abundant carbonate in Earth’s crust, and it is available on all the continents. Relatively to the WMP, Turkey owns 40% of world’s marble reserves, followed by countries such as Italy, Spain, USA, France, Sweden, and Egypt [61]. Portugal, our country, represents 5% of the world’s marble production [15]. The marble processing is accompanied by a considerable amount of waste marble that is generated as a byproduct during the cutting and polishing procedures of the marble rock, which represents about ∼20% of the total marble handled, meaning that each marble producer plant generates a huge number of tons of WMP every year that need to be managed to avoid environmental problems related with its landfill.

## 5. Conclusions

The use of cheap natural materials based on marble powder wastes and natural dolomite as sorbents for CO_2_ capture is extremely appealing to make CaL technology a more sustainable and eco-friendly process.

In this work, the CO_2_ capture performance of natural CaCO_3_-based wastes, such as WMP, a natural dolomite, and a blend of WMP + dolomite, was studied and compared for CaL multiple carbonation–calcination cycles using mild (800 °C, N_2_) and realistic (930 °C, 80% CO_2_) calcination conditions. The synergetic effect of blending WMP and natural dolomite due to the high amount of CaO in the WMP and to the barrier effect provided by the presence of MgO in calcined dolomite was assessed as an approach to tailor cheap wastes-based blended sorbents with improved carrying capacity and stability along the cycles even under realistic industrial calcination conditions.

Under mild calcination conditions, the results show that the deactivation of WMP and dolomite after 20 cycles was 59% and 34%, respectively, and compared to the WMP, a positive effect on the blended sorbent carrying capacity was reached. The results were supported by the textural properties of the sorbents, which evidenced an improved S_BET_ of the blended sorbent comparatively with the WMP, due to the higher MgO content in the blended sorbent (20 vs. 1.1% in calcined basis).

Although in the case of using realistic calcination conditions, the sorbents’ deactivation after 20 cycles is higher when compared with the deactivation observed at mild calcination conditions, promising results were found when using a blended sorbent of WPM + dolomite, since the increase of MgO content in the blended sorbent leads to an improved stability and reactivity along the cycles when compared with WMP used separately as sorbent. The differences of the sorbents carrying capacity, under mild and realistic calcination conditions, can be explained by the CaO and MgO Hüttig temperatures of 679 and 741 °C, respectively, which means that under realistic calcination conditions (930 °C), even for the MgO, this critical temperature is largely exceeded and the sintering process due to surface diffusion is much more relevant.

Since WMP is a waste generated in large amounts in the quarrying marble industry, the use of WMP resources as CaO precursors in blended sorbents with natural dolomite seems a promising and economically attractive option to the circular economy concept and should be encouraged as an eco-friendly material for the CaL process due to the advantages of contributing to reduce the cost of the CaL cycle CO_2_ capture process, as well as to minimize the adverse environmental impacts of the high volume of WMP generated.

## Figures and Tables

**Table 1:** Chemical oxide composition of fresh WMP [15] and dolomite sorbents dried at 120 °C.

Fresh Sorbent	Oxide Content (wt.%)					
	CaO	MgO	Al_2_O_3_	Fe_2_O_3_	SiO_2_	CO_2_
WMP	53.9	0.61	0.11	0.07	1.09	43.6
Dolomite	34.8	17.1	0.02	0.01	0.09	46.2

## Data Availability

Data sharing is not applicable to this article.
